# Topologic connection between 2-D layered structures and 3-D diamond structures for conventional semiconductors

**DOI:** 10.1038/srep24660

**Published:** 2016-04-19

**Authors:** Jianwei Wang, Yong Zhang

**Affiliations:** 1 Department of Electrical and Computer Engineering, The University of North Carolina at Charlotte 9201 University City Boulevard, Charlotte, NC 28223, USA; 2 Microsystem and Terahertz Research Center, China Academy of Engineering Physics 596 Yinhe Road, Shuangliu, Sichuang, 610200, China

## Abstract

When coming to identify new 2D materials, our intuition would suggest us to look from layered instead of 3D materials. However, since graphite can be hypothetically derived from diamond by stretching it along its [111] axis, many 3D materials can also potentially be explored as new candidates for 2D materials. Using a density functional theory, we perform a systematic study over the common Group IV, III–V, and II–VI semiconductors along different deformation paths to reveal new structures that are topologically connected to but distinctly different from the 3D parent structure. Specifically, we explore two major phase transition paths, originating respectively from wurtzite and NiAs structure, by applying compressive and tensile strain along the symmetry axis, and calculating the total energy changes to search for potential metastable states, as well as phonon spectra to examine the structural stability. Each path is found to further split into two branches under tensile strain–low buckled and high buckled structures, which respectively lead to a low and high buckled monolayer structure. Most promising new layered or planar structures identified include BeO, GaN, and ZnO on the tensile strain side, Ge, Si, and GaP on the compressive strain side.

2D materials are the subjects of great current interest. Searching for new 2D materials has been primarily focusing on layered materials, such as graphite, transition metal dichalcogenides, black phosphorus^1^,^2^,^3^. A large number of layered materials have been surveyed theoretically for their potentials becoming new 2D materials^4^. Silicene is perhaps the only noticeable 2D material that is considered as being derived from a 3D structure^5^. Although graphene is commonly viewed as a single layer of a layered material, graphite, there is in fact a topological connection between diamond and graphene: the latter can be viewed as resulting from stretching the former along its [111] axis till the buckling within each bilayer collapses and eventually the bilayers or graphene sheets decouple from each other. This process is illustrated in Fig. 1 along with various other possible related planar or layer structures. By noticing this topological connection, one can envision an alternative avenue for discovering new 2D materials, by exploring wide variety 3D structures of commonly encountered semiconductors. Graphene and BN like 2D materials have been investigated theoretically as monolayers of group IV^6^, III–V^7^, and II–VI (ZnO)^8^. The structural evolution from 3D to layered structures have been studied for C^9^,^10^, BN^11^, BeO^12^, and Si^13^. A related 3D structure NiAs has also been explored for a few binaries: GaN^14^, ZnO^15^, and ZnTe^16^, although the subtle connections among these seemingly very different 3D structures and their topologic connections with different layered structures are not immediately clear. In this work, by examining systematically all the group IV, III–V, and II–VI elemental and binary semiconductors, we offer (1) a comprehensive picture on the topologic connections along different phase transition paths, between a core structure, wurtzite (WZ), and various derivatives: low-buckled and high-buckled layered structures, their asymptotic 2D structures, and NiAs structures; (2) insight to their structural stability and the its dependence on the atomic properties of the elements; (3) predictions for a number of new structures that are potentially achievable experimentally. This work provides guidance to discovering novel 2D materials complementary to those derived from the layered structures, and fundamental insight to the structure-property relationship.

Our basic approach is taking WZ structure as a starting point then searching for other energetically favorable stable or metastable states by stretching and compressing along the c axis of WZ structure, and in addition, allowing one of the bilayers to slip or rotate laterally. A density-functional theory is used for total energy minimization with varying structural parameters. Phonon spectra are calculated to examine the structural stability^17^. A structure with no imaginary phonon mode is deemed (meta) stable against small local distortions.

An evolution tree starting from the WZ structure for a compound XY, with X representing cations and Y anions, is depicted in Fig. 1, illustrating various derivative structures along different deformation paths. The WZ structure is illustrated in Fig. 1(a), where a and c are the lattice parameters in x–y plane and z direction, respectively, and uc is the bond length for the bond along the c axis with u ≈ 3/8. The unit cell contains two monolayers separated by c/2, and each monolayer consists of two closely spaced atomic layers, together known as a bilayer or a buckled single atomic layer. The height of the bilayer or the buckling parameter Δ_WZ_ = (1/2–u)c ≈ c/8 ≈ d_WZ_/3, where d_WZ_ is the bond length. The change in the relative alignment of the two monolayers within one period may result in more variations in structure. There are five distinctly different arrangements:^18^,^19^,^20^,^21^ (1) *AA* stacking (same type atoms in the two monolayers are aligned); (2) 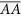 stacking (the opposite type atoms in the two monolayers are aligned, as in WZ ZnO and common h-BN); (3) *A*_X_*B* (cation *X* atoms are aligned, Y atoms are staggered, as in NiAs phase of GaAs); (4) *A*_Y_*B* stacking (anion Y atoms are aligned, X atoms are staggered, also known as anti-NiAs structure), and (5) 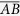 (an one pair of the opposite type atoms in the two monolayers are aligned, but the other pair are staggered). When X and Y are the same, the first two cases will reduce to AA stacked graphite, and the other three cases the common AB stacked graphite. According to these definitions, the WZ structure shown in Fig. 1(a) belongs to 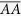, Fig. 1(b) belongs to *A*_X_*B*. Starting from WZ of Fig. 1(a), compressing it along the c axis gives rise to Fig. 1(c) – a planar structure; stretching it leads to Fig. 1(e) – a low buckled (LB) layered structure or Fig. 1(d) – a high buckled (HB) layered structure; rotating or translating one of the monolayers in WZ arrives at Fig. 1(b) – NiAs structure, and further stretching NiAs structure generates a planar or a LB structure Fig. 1(f) or a HB structure Fig. 1(g). If taking the structure of Fig. 1(e) or Fig. 1(f) to a sufficiently large layer separation, one will have a 2D structure that may be either totally flat or slightly buckled, depending on the atomic size. It is implicitly assumed that during structural deformation the atoms are allowed to fully relax to minimize the total energy, but the symmetry about the c axis is kept unchanged. Among all the possible group IV, III–V, and II–VI semiconductors, nine most representative members, including C, Si, Ge, BN, GaN, GaP, BeO, ZnO, and ZnTe, are highlighted in this work.

## Results and Discussion

### Total energy comparison between wurtzite and planar structure

**Figure 1:**
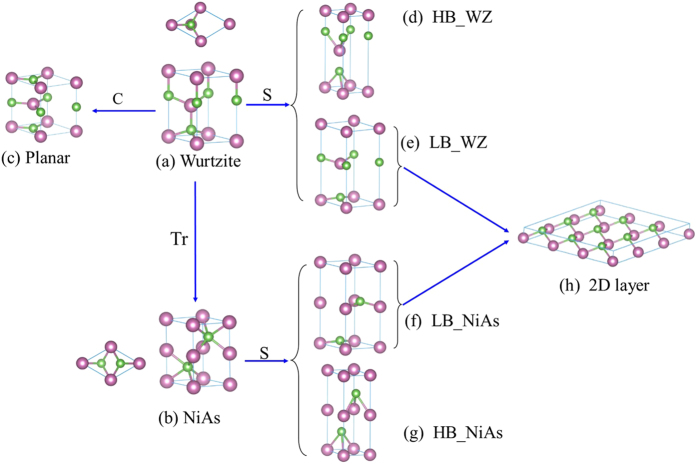
Various structural deformation paths starting from a WZ structure. (**a**) WZ structure; (**b**) NiAs structure by translating (Tr) the upper monolayer of WZ; (**c**) 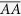 stacking planar structure by compressing (C) the WZ structure; (**d**,**e**), respectively, WZ-HB structure and WZ-LB structure by stretching (S) the WZ structure; (**f**,**g**), respectively, NiAs-LB structure NiAs-HB structure by stretching the NiAs structure; and (**h**) 2D layer structure. The top views of WZ and NiAs structure are also shown next to the respective unit cell.

**Figure 2:**
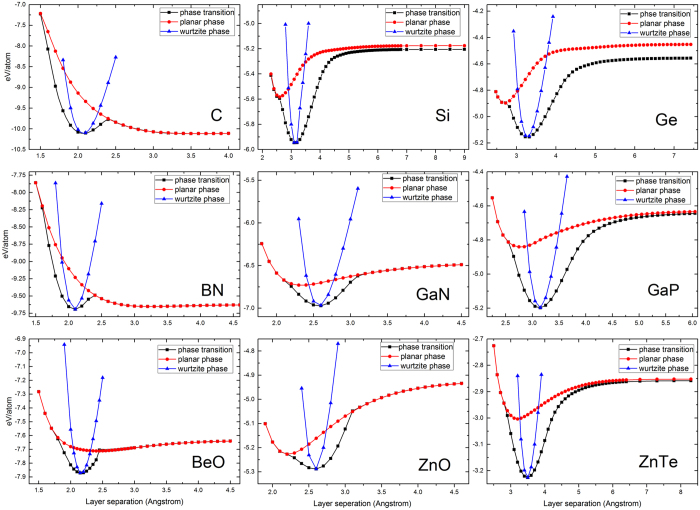
Variation of the total energy vs. c/2 (the separation of the bilayer) for three cases. (**a**) ideal wurtzite structure with a fixed u parameter (blue), (**b**) hexagonal planar structure (red), and (**c**) allowing full relaxation but keeping the 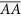 stacking (black).

Among all the group IV, III–V, and II–VI semiconductors, only two of them (C and BN) are known to also have a layered structure consisting of hexagonal planar (HP) layers. The layered structure can be obtained hypothetically, if not always practically feasible, by stretching WZ structure along the c axis, until the buckled bilayers collapse into HP layers (with the nearest neighbor atoms on the same plane), namely AA stacked graphite or h-BN in 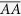 stacking. In a total energy calculation, this phase transition process is manifested as the appearance of a secondary total energy minimum at c_0_^HP^ > c_0_^wz ^9,10,11,22, where c_0_^wz^ and c_0_^HP^ are respectively the c axis lattice constant for the two phases. Such a secondary minimum was not found for other semiconductors that were explored in the past, including Si, GaAs^22^, and BeO^12^. However, we have found that except for C, BN, and BeO, the total energy E_tot,p_(c) curve actually exhibits a secondary minimum with c_0_^HP^ < c_0_^wz^, as shown in Fig. 2, for the rest compounds. The optimized in-plane (*a*) and out-of plane (c) lattice parameters for both the WZ and HP structure are listed in Table 1. Each panel of Fig. 2 includes three curves, respectively calculated for an ideal WZ structure (i.e., with a fixed u value), an ideal 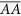 HP structure, and the distorted WZ structure
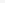
with no constrain on u. Clearly the third curve E_tot_(c) represents the lower-bound of the combined total energy paths described by the other two curves. All these materials fall into one of the two scenarios: (i) c_0_^HP^ > c_0_^wz^, and (ii) c_0_^HP^ <c_0_^wz^. Only C, BN and BeO belong to scenario (i), indicating that they can in principle form a graphite-like planar structure by applying tensile strain. Furthermore, there is an energy barrier between WZ and HP phase in the total energy curve for C, BN and BeO, which helps them to stabilize in either of the two phases. However, BeO has a small barrier of merely 6 meV/atom (missed previously^12^), which is less than 25 meV of room temperature thermal energy. The phonon calculation has revealed that there are imaginary modes for the HP BeO, in contrast to the case for C and BN where no imaginary mode was found. The appearance of imaginary phonon modes indicates that the structure is unstable against distortion that lowers the symmetry. These observations may explain why graphite and h-BN are the only readily available layered materials that are topologically connected to their 3D counterparts. The other six semiconductors of Fig. 2 belong to scenario (ii), implying that a planar phase could in principle appear by applying compressive strain. It may seem counterintuitive that for the material involving large atoms the planar phase would occur at smaller instead of larger c axis layer spacing than that of WZ. In fact, this is because the fact that while some vertical coupling remains a sufficiently large lateral spacing is required to allow the buckled atoms to drop down to the lower plane of the bilayer, depending on the sizes of the atoms involved. Note that the formation of the HP structure on the basal plane does not necessarily mean that the structure has sp^2^ + π bonding or can be considered as a layered material. However, the vertical coupling is expected to be weakened, because even though the c axis lattice constant is reduced, as shown in Fig. 1(c), the separation of the vertical atomic planes has been increased to c_0_^HP^ that is greater than the largest vertical plane separation uc ≈3/8 c_0_^wz^ in the WZ structure. The fact that these planar structures do not produce a secondary minimum on the total energy path E_tot_(c), although it nearly happens for Si, Ge, and ZnO, suggests that the HP phase cannot be a metastable phase of the system in free standing, which is further confirmed by the phonon calculations that yield imaginary modes for these planar structures. However, this finding does not preclude the possibility of forming such planar structure, if the material can be constrained by a proper substrate that can serve as a template for epitaxial growth and provide a weak bonding to the epitaxial layer (ref. 23 for ZnO, ref. 24 for silicene, ref. 25 for monolayer WS_2_). The role of strain in stabilizing graphitic films of InN, AlN, GaN, BeO, ZnO, and SiC has also been studied with first principle calculations^26^.

### Stretching and compressing wurtzite structure

**Figure 3:**
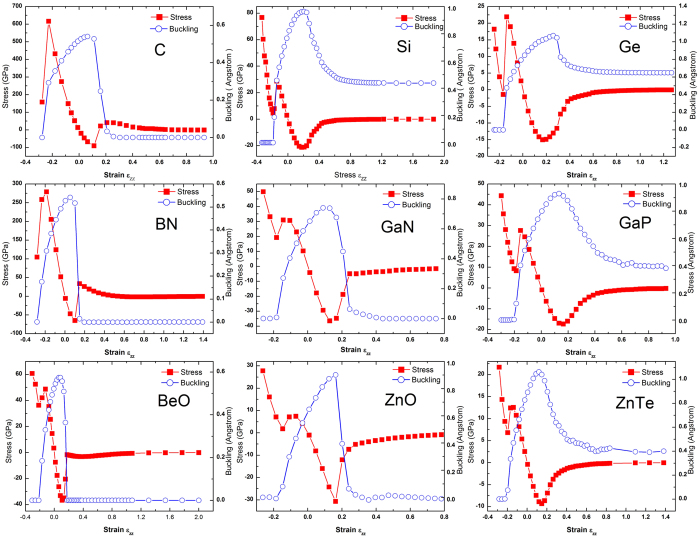
Strain-stress relations and strain-buckling relations for nine semiconductors.

To get more insight about the mechanical and structural properties moving along the total energy curve shown in Fig. 2, the strain-dependent stress X(ɛ_zz_) and buckling height Δ(ɛ_zz_) are calculated and plotted in Fig. 3 for the 9 materials. Taking the WZ structure as a reference, the strain is defined as ɛ_zz _= (c − c_0_^wz^)/c_0_^wz^, and the stress value is obtained directly from the output of the VASP calculation. As shown in Fig. 3, qualitatively, the X(ɛ_zz_) profile is highly asymmetric with respect to ɛ_zz_ = 0. On the compressive strain side (X > 0 and ɛ_zz_ < 0), a large “stress barrier” exists between WZ and HP phase; and after passing the barrier X_B_, the buckling Δ reduces quickly to zero for all the materials, for the reason already given above. On the tensile strain side (ɛ_zz_ > 0), the magnitude of the negative “stress barrier” is usually somewhat smaller than the compressive side. With increasing the c value from c_0_^wz^, the buckling increases initially then quickly reduce to zero for C, BN, GaN, BeO, and ZnO, which all involve at least one first row element, but only to a finite value for Si, Ge, GaP, and ZnTe. For the latter group, as c → ∞, Δ will approach the low-buckled monolayer value reported previously^6^,^7^,^22^. For C, BN, and BeO, after passing the negative “stress barrier”, there appears a zero stress point at finite c, corresponding to the secondary total energy minimum. For the rest materials, X → 0 only as c → ∞, which implies that upon unloading the tensile stress, the material will go back to WZ. For those materials involving the first row elements, one could think of that the buckling is maintained due to the bonding with the atoms in the adjacent bilayer. Thus, as soon as the bonding is weakened to certain extent with increasing the bilayer spacing, the bilayer will collapse spontaneously, because there is adequate lateral space in the lower sub-layer of the bilayer for the atoms in the upper sub-layer to drop down. For those materials involving larger atoms, even the bonding with the other bilayer is fully removed with increasing the bilayer spacing, because the atoms in either the lower or upper sublayer are too big for the atoms of the two sublayers to join each other in the same plane. Thus, compression along the c axis is required to expand the lateral size of the unit cell to make room for the atoms of the upper sub-layer to drop down, while weakening the vertical coupling. Interestingly, such compressed but non-buckled structure actually has a larger interlayer spacing along the c axis. However, the overall atomic density of HP phase is higher than that of WZ. Note that C, BN, and BeO may also reach such a planar structure under compressive strain, but it does not yield a total energy minimum (see Fig. 2).

Quantitatively, for ɛ_zz_ < 0 (compressive strain), for most materials, the “stress barrier” X_B_ is probably too large to be practically achievable to induce a phase transition by compressing the material before shattering it, for instance, X_B_ ≈ 617 GPa and ɛ_zz _= −25.1% for C. However, for ZnO, the barrier height is X_B_ ≈ 7.4 GPa (ɛ_zz_ ≈ −7.0%), and the HP phase appears at ɛ_zz_ = −18.9% and X_HP _≈ 7.1 GPa, which could be practically feasible, keeping in mind that the currently achievable maximum uniaxial stress is about 1 GPa. Although with substantially larger “stress barrier”, the Si and Ge HP phase occur at ɛ_zz_ = −18.9% (X_HP _~ 28.7 GPa) and −16.8% (X_HP _~ 21.9 GPa), respectively. The value of X_B_ and the corresponding strain decreases with increasing atom size, for instance, from C to Si and Ge, which is correlated to the bond length or strength change. The HP phase under uniaxial pressure has been explored theoretically for a number of materials with similar results: AlN^27^, GaN^27^,^28^, BeO^29^, ZnO^23^,^30^,^31^, Zn(S, Se, Te)^31^, MgO^32^, CdS, MgTe, and 2H-SiC^29^. Experimentally, a-few-layer ZnO resembling the HP phase has been observed when deposited on Ag surface^23^, and graphite-like hexagonal nanosheets of AlN have been epitaxially grown through plasma assisted MBE on single crystal Ag(111)^27^. Additionally, it was predicted that a WZ ZnO nanowire would undergo a phase transition to the HP phase (called HX phase) either being compressed or stretched^33^. For ɛ_zz_ > 0 (tensile strain), ZnTe is found to have the smallest barrier |X_B_| ≈ 9.3 GPa (ɛ_zz _≈ 14.8%). For ZnO, |X_B_| ≈ 30.7 GPa (ɛ_zz _≈ 16.3%), and Δ → 0 at ɛ_zz _≈ 31.8% (|X_B_| ≈ 4.5 GPa). This result suggests that it is unlikely practically feasible to stretch a ZnO nanowire to the point of Δ → 0. Our result also contradicts to the previous prediction about the possibility to obtain the HX phase (with c_0_ < c_0_^WZ^) under tensile loading^33^. Note that the magnitude of |X_B_| indicates how easy one could deform WZ structure to reach the planar structure either on the compressive or tensile side, whereas |X_HP_| reflects the easiness of forming the structure by providing some external constrain, such as through epitaxial growth.

For simplicity, we have adopted the WZ structure instead of ZB as the starting point. For the tensile strain side, the small difference at the starting point between WZ and ZB will not affect the final 2D material, although the intermediate states could be somewhat different. For the compressive strain side, the difference might be more significant. Therefore, our results should be understood as one possible scenario, and the evolution starting from the ZB structure should be studied separately.

### Global total energy search for metastable states

Thus far, we have assumed that during either compressing or stretching WZ structure there is no lateral motion of atoms within each layer. Here we show relaxing this constrain will lead to various other structures. As illustrated in Fig. 1, WZ structure is the starting point from a structure with a buckled basal plane to evolve into planar basal plane by either stretching or compressing along the c axis. All these structures belong to the category of 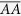 stacking. For the single element material C, 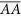 is the same as AA, and the corresponding structure will be the AA stacked graphite that does not occur naturally but can be obtained under certain conditions (ref. 34 and refs therein). Similarly, we can explore the possible phase transitions for the AB stacked structures, by taking NiAs structure as the starting point then either stretching or compressing along the c axis. NiAs is also a buckled structure on the basal plane, as shown in Fig. 1(b), which is topologically connected to WZ structure. For the single element material C, stretching a NiAs phase C, although non-existing either in reality or theoretically, will directly lead to the familiar AB stacked or common graphite phase.

**Figure 4:**
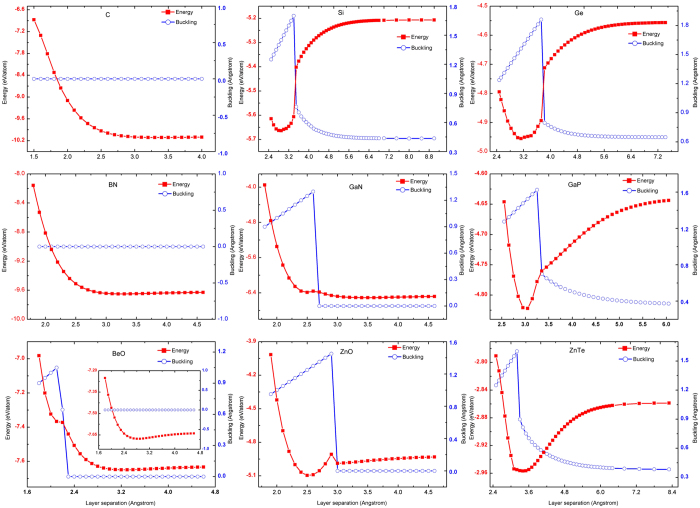
Phase transition path around NiAs-like structure. The line with red squares indicates the total energy vs. layer separation and the line with blue circles indicates the buckling height vs. layer separation.

For a given compound XY, among the three possible AB stacked structures: (1) *A*_X_*B*, (2) *A*_Y_*B* stacking, and (3) 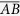. 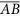 tends to be more energetically favorable over *A*_X_*B* or *A*_Y_*B*. For instance, for GaN and BN, both 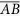 and *A*_X_*B* are shown to be stable but *A*_Y_*B* is unstable from the phonon calculations, agreeing with previous findings for GaN^21^ and h-BN^18^,^19^,^20^. The total energy based phase transition path for the 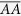 structure has been explored in Fig. 2, now we investigate the similar phase transition path as well as the variation in buckling parameter for the *A*_X_*B* structure for the same 9 materials with the results shown in Fig. 4. The obtained structure parameters are summarized in Table 2, with comparison to the available literature values. They can be divided into three groups: (i) C and BN with only one total energy minimum, corresponding to the common graphite and one of the known h-BN phases (Δ = 0). (ii) BeO, ZnO, and GaN with two total energy minima that correspond to the NiAs (Δ ≠ 0) and planar phase (Δ = 0), respectively. For BeO and GaN, the NiAs phase has a higher total energy than the planar, whereas for ZnO, the NiAs phase is lower. (iii) Si, Ge, GaP, and ZnTe with only one total energy minimum, corresponding to the NiAs phase. The NiAs phase has previously been studied for GaN^14^, ZnO^15^, and ZnTe^16^, but the structural stability has not been explicitly examined. In contrast to a previous report suggesting the existence of BN in NiAs phase^35^, we do not find NiAs phase for BN. We have performed phonon calculations for the NiAs phase and planar phase, and found that: (1) the NiAs phase is stable for Si, Ge, GaN, GaP, and ZnO, but not for BeO; (2) the *A*_X_*B* planar phase (p-*A*_X_*B*) is stable for C and BN, as is known; however, for GaN, ZnO, and BeO, even though there is a secondary total energy minimum point, the *A*_X_*B* planar structure is unstable, judging by the existence of imaginary phonon modes. (3) Nevertheless, BeO in 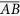 stacking, p-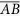, is a stable planar phase, which has not been reported before, as shown as an inset of the BeO panel in Fig. 4; and (4) some imagine modes were found for the NiAs phase ZnTe. In short, NiAs phase is only stable for binaries with moderate size atoms; p-*A*_X_*B* is only stable for binaries with small size atoms; neither NiAs nor p-*A*_X_*B* is stable for BeO, but 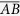 is.

We next discuss the variation of the buckling height with changing the bilayer separation starting from the NiAs phase. On the tensile strain side, the dependence is very similar to that shown in Fig. 3 for the phase transition curve starting from WZ structure. With increasing layer separation, the buckling heights remain or tend to zero for C, BN, BeO, GaN, and ZnO, but to lower values for Si, Ge, GaP, and ZnTe, i.e., the bilayer only collapses partially. The presence of the first row elements N and O make it easier to form a planar structure. However, on the compressive strain side, the layer-separation dependence of the buckling height is rather different with those shown in Fig. 3 starting from WZ structure. Excluding C and BN that do not exhibit the NiAs phase, all the other seven retain the NiAs structures under compression, i.e., the buckling height decreases proportionally with the layer separation but always equals to 0.25c. The different response between NiAs and WZ structure lies in that the AB stacking has a closer packing thus more robust against the compression than the AA stacking, because in AB one atom is staggered over another, thus it is easier to form a close packed structure. The effect of this close packed structure can be seen from the fact that the c axis lattice parameter has a larger value in WZ than in NiAs, and it is true even for the in-plane lattice constant. Taking Si as an example, the c value is 6.29 Å vs. 5.636 Å, and the *a* axis lattice constant 3.80 Å vs. 3.587 Å.

### Comprehensive examination of Si

The possible phase transitions starting from WZ and NiAs-like structures have been discussed respectively in the previous sections. However, there are other possibilities beyond those that have already been included in Figs 2 and 4. It has been shown for a monolayer structure, in addition to the planar or low buckled (LB) structure, there is a high buckled (HB) structure that sometime even has a lower total energy than the former. Here LB and HB structures, respectively, have a lower and higher buckling height compared to the WZ structure. However, the HB structure has been found to be unstable, based on the phonon calculation^6^. It is unclear how this HB structure is related to the known 3D structures, either WZ or NiAs phase. Here we use Si as an example to offer a more comprehensive examination over the possible structures that could be derived from the 3D structures. Because involving only one element, the five possible stacking orders have been reduced to two, i.e., AA and AB, which respectively corresponds to WZ and NiAs structure.

**Figure 5:**
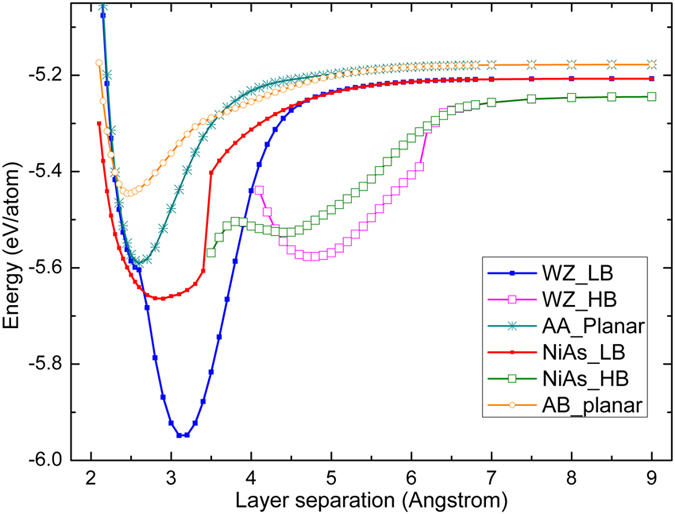
Phase transition curves for silicon with either wurtzite or NiAs structure (including high and low bucking), and planar structures with AA and AB stacking.

Shown in Fig. 5 are the phase transition curves under compression and expansion starting from either WZ or NiAs phase for Si. In addition, two curves for planar AA and AB structures are also included. One significant feature is that under tensile strain, for either the WZ or NiAs curve, the transformation curve actually splits into two branches: one corresponding to the LB structure that has been shown, respectively, in Figs 2 and 4; the other branch, which was not shown there, corresponding to a HB structure that has not been identified before. After the splitting point, the HB branch in fact has lower energy. For the WZ curve, the HB structure has a total energy minimum at c = 4.8 Å, and the curve in fact extrapolates to the HB monolayer structure reported previously^6^. For the NiAs curve, the HB minimum occurs at c = 4.4 Å. Unfortunately, neither of these minima yields a metastable state, based on the appearance of imaginary phonon modes. At c → ∞, the two LB branches merge to the same point – silicene, which is structurally stable^6^, although not necessary chemically stable (i.e., easily reacting with other species). Similarly, at c → ∞, the two HB curves merge together, but none of them is structurally stable. The curve for the AB stacking planar structure is found to have a minimum at c = 5.2 Å, which actually corresponds to the “graphitic” Si reported earlier^13^. Again, this is not a metastable state from the phonon calculation, but nevertheless might be possible to achieve if constrained by a proper substrate. The energy minimum of planar structure with AB stacking is higher than that of planar structure with AA stacking, which is similar to the case that the energy of the NiAs phase is higher than the WZ phase. However, there is an energy barrier between them.

We have offered a bird’s-eye view of the topological connections between the 2-D structure, layered or planar structure, and 3-D wurtzite or NiAs structure. Stretching or compressing the 3D structure along the WZ or NiAs c axis will lead to each monolayer of the 3D structure to either fully or partially collapse into a planar or quasi-planar layer, resulting in a new intermediate structure with stacked planar or quasi-planar layers. These intermediate structures are the candidates for developing new 2D materials, if the planar or quasi-planar layers can be individually isolated. Among the nine representative Group IV, III-V, and II-VI 3D semiconductors that have been explicitly investigated for the described structural evolution, eight intermediate structures have been identified to be of particularly interest: (1) BeO has a graphite-like (with 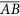 stacking) metastable structure, which can exist in free-standing. (2) Four graphite-like planar structures (with c_p_ > c_3D_) which are metastable structures based on total energy consideration, although unstable against vibrational distortion, including BeO in 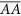 stacking derived from WZ phase, BeO, ZnO, and GaN in A_X_B stacking derived from NiAs phase. However, these structures might be achievable by epitaxial growth on proper substrates either as single or multiple monolayers. (3) Three planar structures of Ge, Si, and GaP in 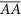 stacking derived from WZ phase, which are close to have a total energy minimum (with c_p_ < c_3D_). It might be possible to grow these unstable structures epitaxially on proper substrates. A single or few monolayer(s) of these graphite-like structures could be the new candidates of 2D materials for exploration. In addition, the NiAs structure has been predicted to be a metastable state for Si, Ge, and GaP. In this study, we have investigated the feasibility how a monolayer in the [0001] direction of WZ structure or [111] direction of ZB structure can be modified to form a (quasi-) 2D layer, because of a clear topologic connection between the 3D structure and the graphene-like 2D hexagonal structure. However, a less trivial quasi-2D structure may also be derived from a two-monolayer (110) slab of ZB or (11
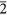
0) slab of WZ by imaging to continuously cleave the ZB structure along the (110) plane or perhaps more realistically by “intercalating” the two-monolayer slabs with organic molecules^36^,^37^. Looking beyond the database of the conventional layered materials, a plenty of 3D materials could be explored as new 2D systems.

## Methods

The total energy calculations are performed by using a pseudopotential density functional (DFT) method implemented in VASP code^38^. The projector augmented plane-wave (PAW) method within a local density approximation (LDA)^39^, and k mesh of 12 × 12 × 8 are employed in the total energy calculation. Phonon calculations were performed using PHONOPY code^17^ 17 by a supercell approach with the supercell size of 3 × 3 × 2 unit cells, where a set of supercells generated through a finite displacement method. The force constants for the supercell were calculated using VASP code, and phonon frequencies were calculated from the force constants using PHONOPY code. The electronic and phonon dispersion curves for a few key structures are given in Supplemental Materials.

## Additional Information

**How to cite this article**: Wang, J. and Zhang, Y. Topologic connection between 2-D layered structures and 3-D diamond structures for conventional semiconductors. *Sci. Rep*. **6**, 24660; doi: 10.1038/srep24660 (2016).

## Supplementary Material

Supplementary Information

## Figures and Tables

**Table 1:** Optimized structure parameters for nine semiconductors with wurtzite (WZ) and 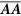 hexagonal planar phase ( 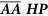 ).

		Wurtzite phase					Hexagonal planar phase			
		This work		Previous work			This work		Previous work	
	E_tot_ (eV)	a (Å)	c (Å)	a (Å)	c (Å)	E_tot_(eV)	a (Å)	c (Å)	a (Å)	c (Å)
C	−10.109	2.49	4.14	2.51^a^	4.13^a^	−10.116	2.45	7.22	2.44^j^	7.22^j^
Si	−5.937	3.80	6.29	3.84^b^	6.28^b^	−5.583	4.09	5.03	4.07^k^	4.95^k^
Ge	−5.157	3.98	6.55	3.96^c^	6.57^c^	−4.899	4.36	5.39		
BN	−9.695	2.52	4.18	2.558^d^	4.423^d^	−9.653	2.490	6.488	~2.49^m^	~6.48^m^
GaN	−6.973	3.16	5.14	3.190^e^	5.189^e^	−6.731	3.279	4.682		
GaP	−5.198	3.80	6.27	3.8419^f^	6.3353^f^	−4.841	4.011	5.576		
BeO	−7.872	2.667	4.333	2.698^g^	4.377^g^	−7.712	2.679	4.844		
ZnO	−5.289	3.17	5.16	3.250^h^	5.201^h^	−5.227	3.378	4.436	3.34^n^	4.32^n^
ZnTe	−3.226	4.23	6.97	4.234^i^	6.978^i^	−3.004	4.432	6.386		

^a^Ref. 40. ^b^Ref. 41. ^c^Ref. 42.

^d^Ref. 43. ^e^Ref. 44. ^f^Ref. 45.

^g^Ref. 46. ^h^Ref. 47. ^i^Ref. 16.

^j^Ref. 24. ^k^Ref. 13. ^m^Ref. 19.

^n^Ref. 23.

Calculated values for total energy/atom are also given. WZ is (meta)stable for all, HP is only (meta)stable for C and BN.

**Table 2:** Optimized A_X_B stacking structure parameters of nine semiconductors.

	E_tot_(eV)	Present work		u	Previous results		Structure
		a (Å)	c (Å)		a (Å)	c (Å)	
C	−10.127	2.446	6.643	0	2.4395^a^	6.6589^a^	p-A_X_B (*)
Si	−5.664	3.587	5.636	0.25			NiAs (*)
Ge	−4.956	3.775	6.226	0.25			NiAs (*)
BN	−7.792	2.466	3.979	0			NiAs
	−9.651	2.489	6.469	0			p-A_X_B (*)
GaN	−6.391	2.945	4.997	0.25	3.02^b^	5.07^b^	NiAs (*)
	−6.492	3.147	6.426	0			p-A_X_B
GaP	−4.824	3.532	6.014	0.25			NiAs (*)
BeO	−7.372	2.491	4.180	0.25			NiAs
	−7.655	2.641	6.439	0			p-A_X_B
	−7.677	2.650	5.699	0			p- 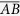 (*)
ZnO	−5.097	2.919	5.028	0.25	2.94^c^	5.07^c^	NiAs (*)
	−4.982	3.196	5.372	0			p-A_X_B
ZnTe	−2.967	3.941	6.552	0.25	3.956^d^	6.563^d^	NiAs

Calculated values for total energy/atom are also given. Metastable structures are indicated with “*”.

^a^Ref. 24.

^b^Ref. 14.

^c^Ref. 15

^d^Ref. 16.

## References

[b1] Xu M., Liang T., Shi M. & Chen H. Graphene-Like Two-Dimensional Materials. Chem. Rev. 113, 3766–3798 (2013). 23286380 10.1021/cr300263a

[b2] Butler S. Z. . Progress, Challenges, and Opportunities in Two-Dimensional Materials Beyond Graphene. ACS Nano 7, 2898–2926 (2013). 23464873 10.1021/nn400280c

[b3] Koski K. J. & Cui Y. The New Skinny in Two-Dimensional Nanomaterials. ACS Nano 7, 3739–3743 (2013). 23678956 10.1021/nn4022422

[b4] Lebègue S. . Two-Dimensional Materials from Data Filtering and Ab Initio Calculations. Phys. Rev. X 3, 031002 (2013).

[b5] Guzmán-Verri G. G. & Lew Yan Voon L. C. Electronic structure of silicon-based nanostructures. Phys. Rev. B 76, 075131 (2007).

[b6] Cahangirov S. . Two- and One-Dimensional Honeycomb Structures of Silicon and Germanium. Phys. Rev. Lett. 102, 236804 (2009). 19658958 10.1103/PhysRevLett.102.236804

[b7] Şahin H. . Monolayer honeycomb structures of group-IV elements and III–V binary compounds: First-principles calculations. Phys. Rev. B 80, 155453 (2009).

[b8] Topsakal M., Cahangirov S., Bekaroglu E. & Ciraci S. First-principles study of zinc oxide honeycomb structures. Phys. Rev. B 80, 235119 (2009).

[b9] Fahy S., Louie S. G. & Cohen M. L. Pseudopotential total-energy study of the transition from rhombohedral graphite to diamond. Phys. Rev. B 34, 1191–1199 (1986). 10.1103/physrevb.34.1191 9939737

[b10] Fahy S., Louie S. G. & Cohen M. L. Theoretical total-energy study of the transformation of graphite into hexagonal diamond. Phys. Rev. B 35, 7623–7626 (1987). 10.1103/physrevb.35.7623 9941068

[b11] Wentzcovitch R. M., Fahy S., Cohen M. L. & Louie S. G. Ab initio study of graphite → diamondlike transitions in BN. Phys. Rev. B 38, 6191–6195 (1988). 10.1103/physrevb.38.6191 9947080

[b12] Continenza A., Wentzcovitch R. M. & Freeman A. J. Theoretical investigation of graphitic BeO. Phys. Rev. B 41, 3540–3544 (1990). 10.1103/physrevb.41.3540 9994150

[b13] Wang Y., Scheerschmidt K. & Gösele U. Theoretical investigations of bond properties in graphite and graphitic silicon. Phys. Rev. B 61, 12864–12870 (2000).

[b14] Muoz A. & Kunc K. High-pressure phase of gallium nitride. Phys. Rev. B 44, 10372–10373 (1991). 10.1103/physrevb.44.10372 9999052

[b15] Zagorac D., Schön J. C., Zagorac J. & Jansen M. Prediction of structure candidates for zinc oxide as a function of pressure and investigation of their electronic properties. Phys. Rev. B 89, 075201 (2014).

[b16] Yang J.-H. . Electronic structure and phase stability of MgTe, ZnTe, CdTe, and their alloys in the B3, B4, and B8 structures. Phys. Rev. B 79, 245202 (2009).

[b17] Togo A., Oba F. & Tanaka I. First-principles calculations of the ferroelastic transition between rutile-type and CaCl_2_-type SiO_2_ at high pressures. Phys. Rev. B 78, 134106 (2008).

[b18] Liu L., Feng Y. P. & Shen Z. X. Structural and electronic properties of h-BN. Phys. Rev. B 68, 104102 (2003).

[b19] Ooi N., Rairkar A., Lindsley L. & Adams J. B. Electronic structure and bonding in hexagonal boron nitride. J. Phys: Condens. Matter 18, 97 (2006).

[b20] Constantinescu G., Kuc A. & Heine T. Stacking in Bulk and Bilayer Hexagonal Boron Nitride. Phys. Rev. Lett. 111, 036104 (2013). 23909342 10.1103/PhysRevLett.111.036104

[b21] Tsipas P. . Evidence for graphite-like hexagonal AlN nanosheets epitaxially grown on single crystal Ag(111). Appl. Phys. Lett. 103, 251605 (2013).

[b22] Miguel F.-C. . Theoretical study of graphitic analogues of simple semiconductors. Modell. Simul. Mater. Sci. Eng. 7, 929 (1999).

[b23] Tusche C., Meyerheim H. L. & Kirschner J. Observation of Depolarized ZnO(0001) Monolayers: Formation of Unreconstructed Planar Sheets. Phys. Rev. Lett. 99, 026102 (2007). 17678236 10.1103/PhysRevLett.99.026102

[b24] Zhang Y. & Tsu R. Binding Graphene Sheets Together Using Silicon: Graphene/Silicon Superlattice. Nanoscale Res. Lett. 5, 805–808 (2010). 20672119 10.1007/s11671-010-9561-x PMC2893836

[b25] Su L., Yu Y., Gao L. & Zhang Y. High Temperature Behavior of Monolayer WS2 and Its Interaction with Substrate: Dependence on Substrate Type and Bonding. Nano Res. 8, 2686–2697 (2015).

[b26] Wu D., Lagally M. G. & Liu F. Stabilizing Graphitic Thin Films of Wurtzite Materials by Epitaxial Strain. Phys. Rev. Lett. 107, 236101 (2011). 22182104 10.1103/PhysRevLett.107.236101

[b27] Schwierz F., Pezoldt J. & Granzner R. Two-dimensional materials and their prospects in transistor electronics. Nanoscale 7, 8261–8283 (2015). 25898786 10.1039/c5nr01052g

[b28] Dong L., Yadav S. K., Ramprasad R. & Alpay S. P. Band gap tuning in GaN through equibiaxial in-plane strains. Appl. Phys. Lett. 96, 202106 (2010).

[b29] Shi L. . Strain-assisted structural transformation and band gap tuning in BeO, MgTe, CdS and 2H-SiC: A hybrid density functional study. Europhys. Lett. 106, 57001 (2014).

[b30] Freeman C. L., Claeyssens F., Allan N. L. & Harding J. H. Graphitic Nanofilms as Precursors to Wurtzite Films: Theory. Phys. Rev. Lett. 96, 066102 (2006). 16606013 10.1103/PhysRevLett.96.066102

[b31] Yadav S. K., Sadowski T. & Ramprasad R. Density functional theory study of ZnX (X = O, S, Se, Te) under uniaxial strain. Phys. Rev. B 81, 144120 (2010).

[b32] Goniakowski J., Noguera C. & Giordano L. Using Polarity for Engineering Oxide Nanostructures: Structural Phase Diagram in Free and Supported MgO(111) Ultrathin Films. Phys. Rev. Lett. 93, 215702 (2004). 15601031 10.1103/PhysRevLett.93.215702

[b33] Kulkarni A. J., Zhou M., Sarasamak K. & Limpijumnong S. Novel Phase Transformation in ZnO Nanowires under Tensile Loading. Phys. Rev. Lett. 97, 105502 (2006). 17025826 10.1103/PhysRevLett.97.105502

[b34] Yue N., Zhang Y. & Tsu R. Ambient condition laser writing of graphene structures on polycrystalline SiC thin film deposited on Si wafer. Appl. Phys. Lett. 102, 071912 (2013).

[b35] Hromadová L. & Martoňák R. Pressure-induced structural transitions in BN from ab initio metadynamics. Phys. Rev. B 84, 224108 (2011).

[b36] Inoshita T., Jeong S., Hamada N. & Hosono H. Exploration for Two-Dimensional Electrides via Database Screening and Ab Initio Calculation. Phys. Rev. X 4, 031023 (2014).

[b37] Sun Y. . Fabrication of flexible and freestanding zinc chalcogenide single layers. Nat. Commun. 3, 1057 (2012). 22968703 10.1038/ncomms2066

[b38] Xu D., He H., He Pandey, R P. & Karna S. Stacking and electric field effects in atomically thin layers of GaN. J. Phys: Condens. Matter 25, 345302 (2013). 23896638 10.1088/0953-8984/25/34/345302

[b39] Kresse G. & Hafner J. Ab initio molecular dynamics for liquid metals. Phys. Rev. B 47, 558–561 (1993). 10.1103/physrevb.47.558 10004490

[b40] Bundy F. P. & Kasper J. S. Hexagonal Diamond–A New Form of Carbon. J. Chem. Phys. 46, 3437–3446 (1967).

[b41] Zhang Y., Iqbal Z., Vijayalakshmi S. & Grebel H. Stable hexagonal-wurtzite silicon phase by laser ablation. Appl. Phys. Lett. 75, 2758–2760 (1999).

[b42] Xiao S.-Q. & Pirouz P. On diamond-hexagonal germanium. J. Mater. Res. 7, 1406–1412 (1992).

[b43] Sōma T., Sawaoka A. & Saito S. Characterization of wurtzite type boron nitride synthesized by shock compression. Mater. Res. Bull. 9, 755–762 (1974).

[b44] Schulz H. & Thiemann K. H. Crystal structure refinement of AlN and GaN. Solid State Commun. 23, 815–819 (1977).

[b45] Kriegner D. . Unit cell structure of the wurtzite phase of GaP nanowires: X-ray diffraction studies and density functional theory calculations. Phys. Rev. B 88, 115315 (2013).

[b46] Hazen R. M. & Finger L. W. High‐pressure and high-temperature crystal chemistry of beryllium oxide. J. Appl. Phys. 59, 3728–3733 (1986).

[b47] Decremps F. . Local structure of condensed zinc oxide. Phys. Rev. B 68, 104101 (2003).

