# Supplementary material for: Topologic connection between 2-D layered structures and 3-D diamond structures for conventional semiconductors
Source: Sci Rep. 2016 Apr 19;6:24660. doi: 10.1038/srep24660 (PMC4835777; doi:10.1038/srep24660)
Supplement: Supplementary Information [file srep24660-s1.pdf]

Supplemental Material

Topologic connection between 2-D layered structures and 3-D diamond structures for conventional semiconductors

Jianwei Wang[1,2] and Yong Zhang[1,*]

[1]Department of Electrical and Computer Engineering,The University of North Carolina at Charlotte

9201 University City Boulevard, Charlotte, NC 28223, USA

[2]Microsystem and Terahertz Research Center, China Academy of Engineering Physics,

596 Yinhe Road, Shuangliu, Sichuang, 610200, China

Electronic and phonon dispersion curves for a few key structures

(1) BN: $A_{\mathrm{X}}B$ stacking planar structure

(2) BN: $\overline{AB}$ stacking planar structure

(3) GaP: NiAs structure (newly identified)

(4) Ge: NiAs structure (newly identified)

(5) Si: NiAs structure (newly identified)

(6) ZnO: NiAs structure

(7) GaN: NiAs structure

(8) BeO: $\overline{AB}$ stacking planar structure (newly identified)

* Correspondence: yong.zhang@uncc.edu

(1) BN in $A_X B$ stacking planar structure

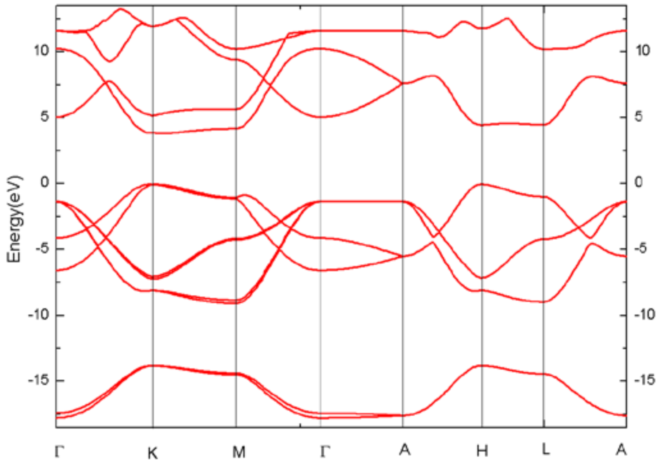

(a) Electronic structure.

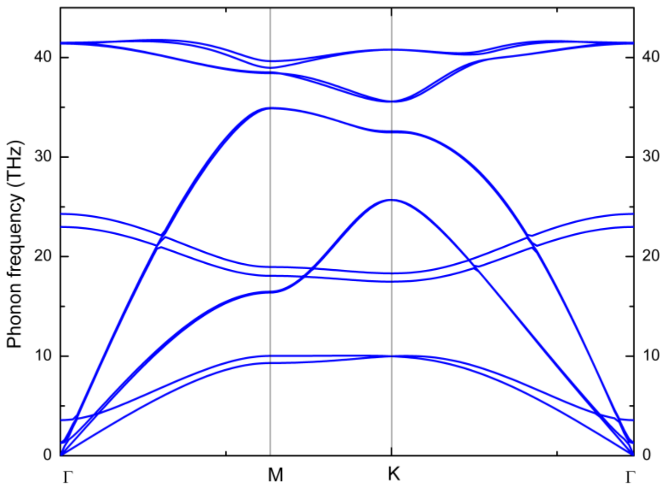

(b) Phonon dispersion curves.

(2) BN: $\overline{AB}$ stacking planar structure

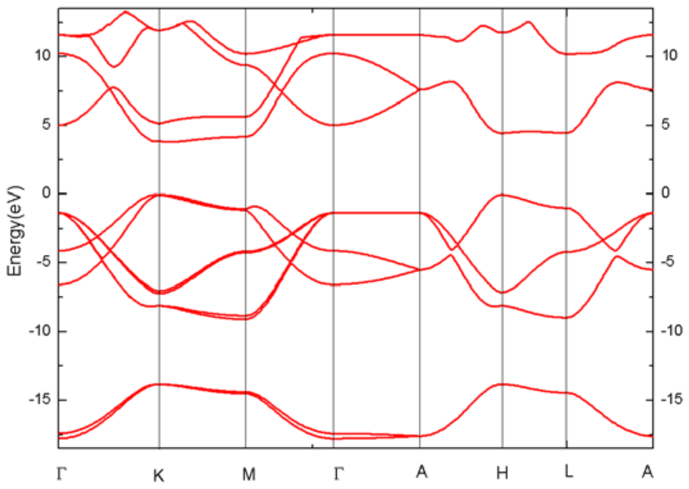

(a) Electronic structure.

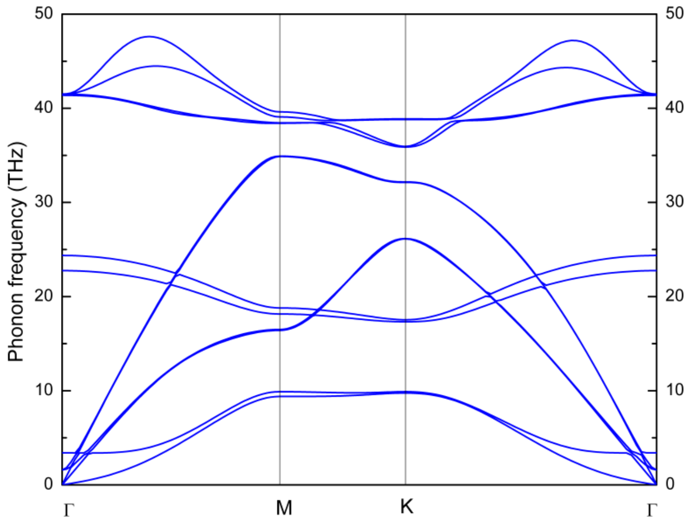

(b) Phonon dispersion curves.

(3) GaP: NiAs structure (newly identified)

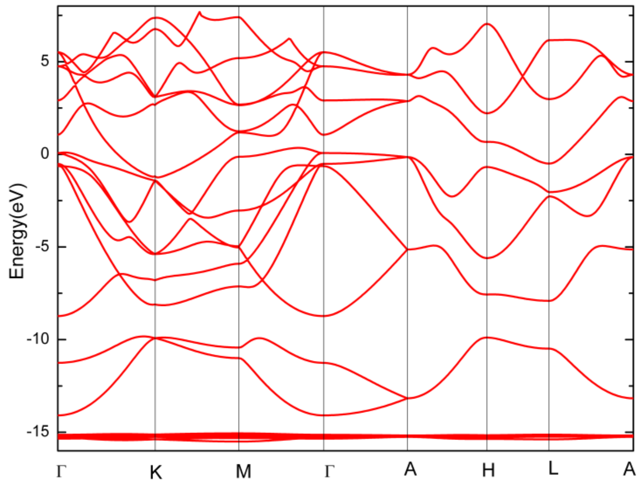

(a) Electronic structure.

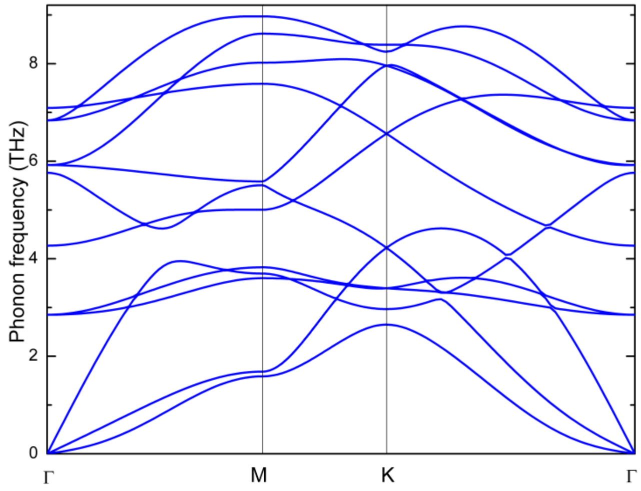

(b) Phonon dispersion curves.

(4) Ge: NiAs structure (newly identified)

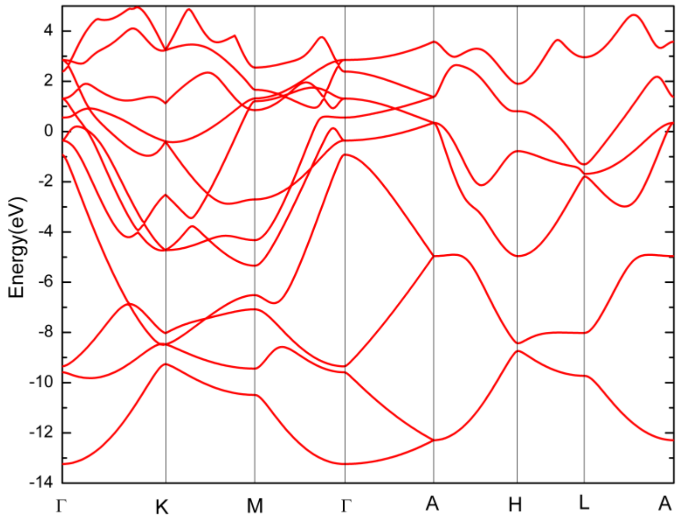

(a) Electronic structure.

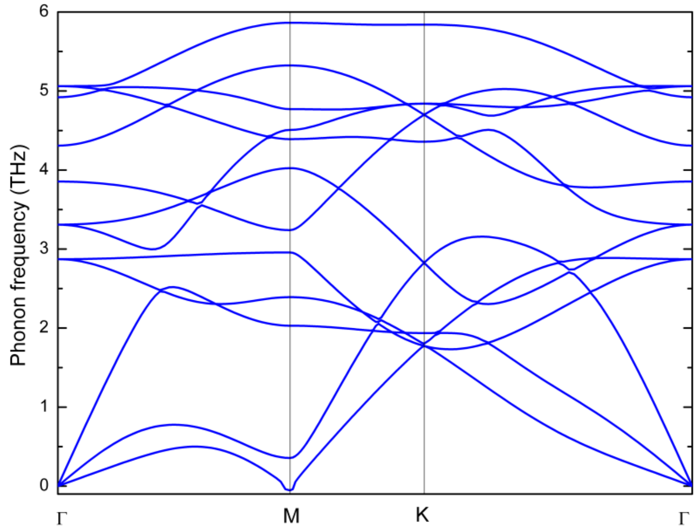

(b) Phonon dispersion curves.

(5) Si: NiAs structure (newly identified)

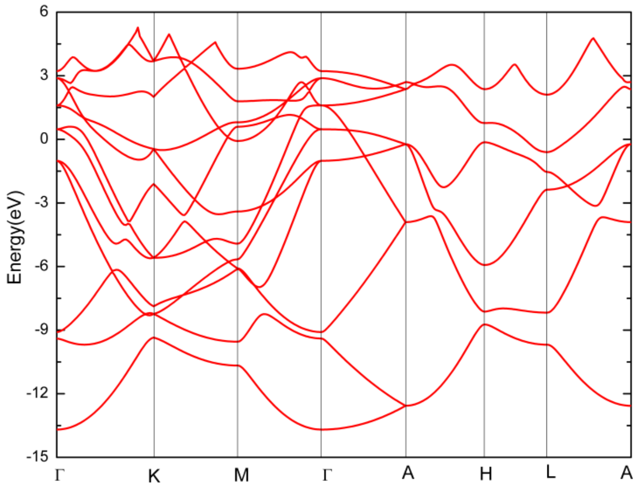

(a) Electronic structure.

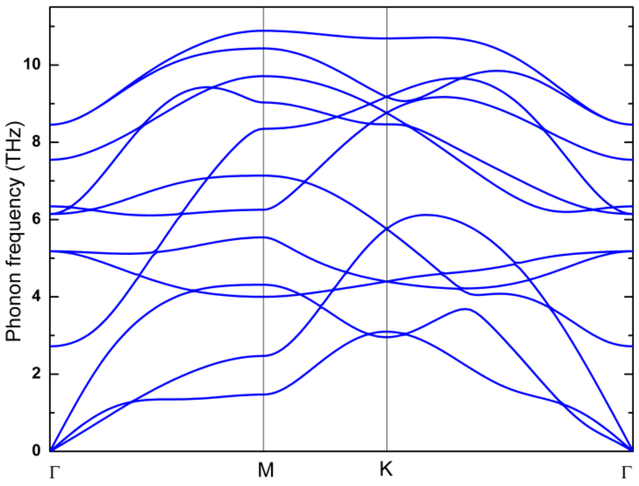

(b) Phonon dispersion curves.

(6) ZnO: NiAs structure

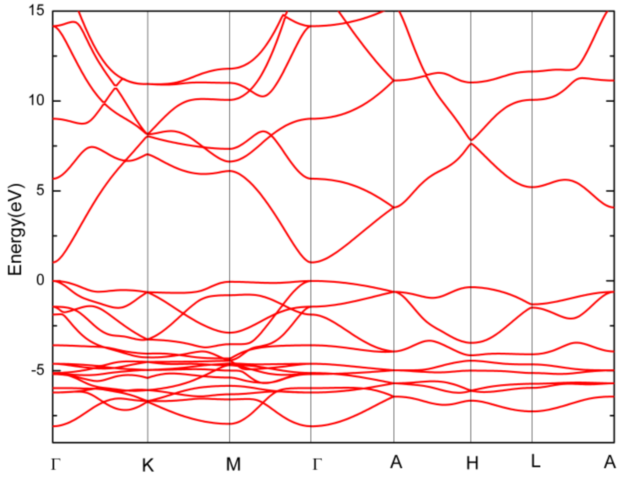

(a) Electronic structure.

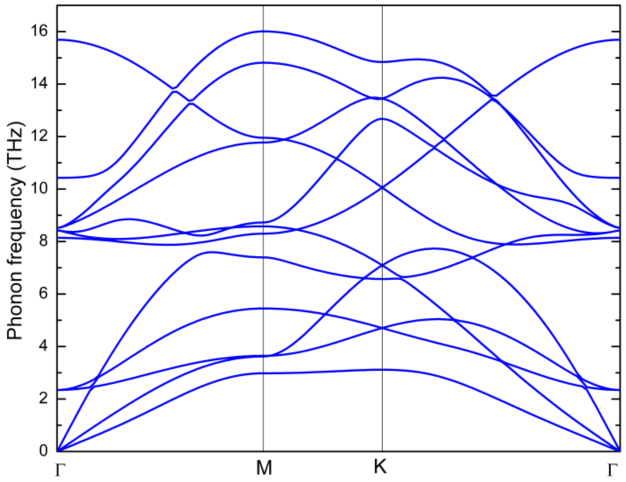

(b) Phonon dispersion curves.

(7) GaN: NiAs structure

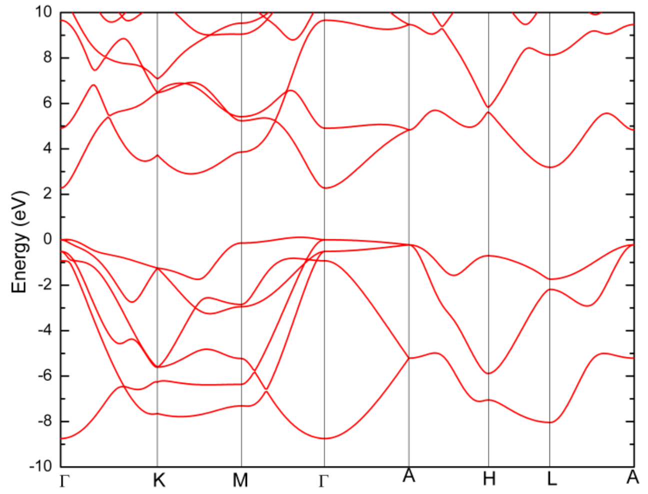

(a) Electronic structure.

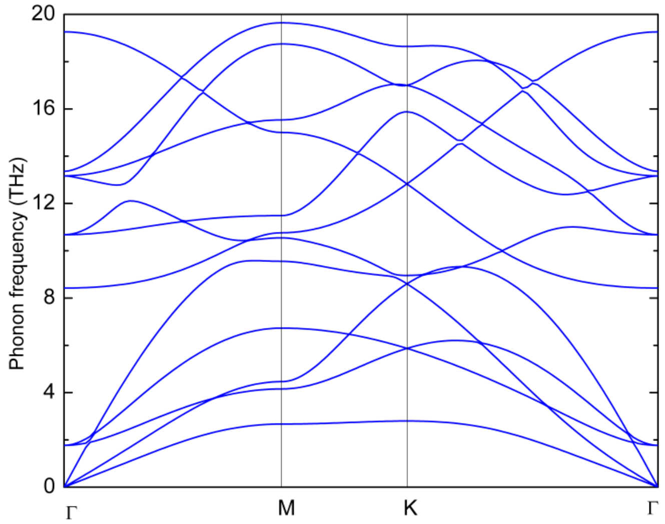

(b) Phonon dispersion curves.

(8) BeO: $\overline{AB}$ stacking planar structure (newly identified)

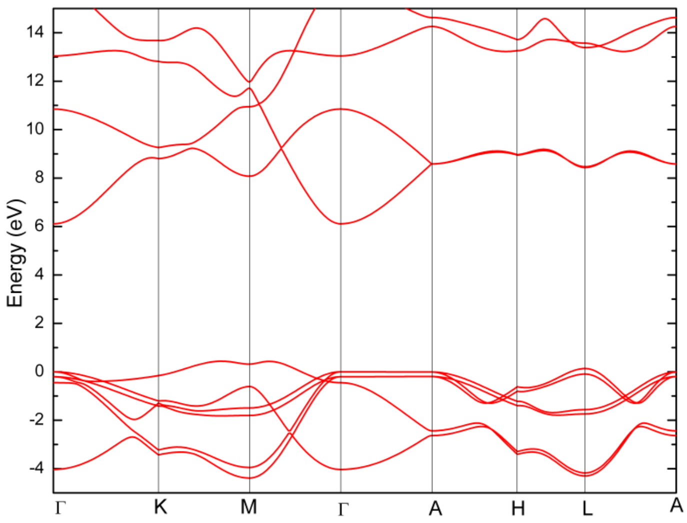

(a) Electronic structure.

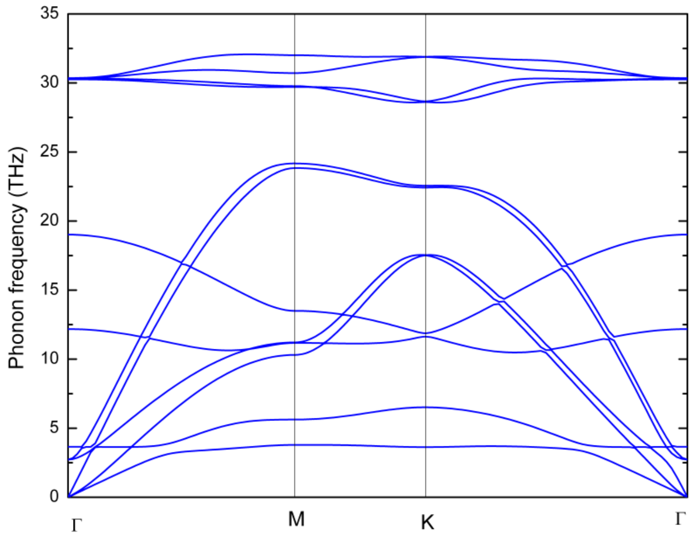

(b) Phonon dispersion curves.
